# What Works? the Influence of Changing Wastewater Treatment Type, Including Tertiary Granular Activated Charcoal, on Downstream Macroinvertebrate Biodiversity Over Time

**DOI:** 10.1002/etc.4460

**Published:** 2019-07-30

**Authors:** Andrew C. Johnson, Monika D. Jürgens, François K. Edwards, Peter M. Scarlett, Helen M. Vincent, Peter von der Ohe

**Affiliations:** ^1^ Centre for Ecology & Hydrology Wallingford United Kingdom; ^2^ Amalex Environmental Solutions Leipzig Germany

**Keywords:** Wastewater, River, Water quality, Macroinvertebrate, Sensitivity, Biodiversity

## Abstract

The present study reviewed the impacts of wastewater on macroinvertebrates over 4 decades in a United Kingdom lowland river. This involved examining changes in chemicals, temperature, flow, and macroinvertebrate diversity from the 1970s until 2017 for a wastewater‐dominated river downstream of Swindon in the United Kingdom (population ~ 220 000). When the wastewater treatment process changed from trickling filter to activated sludge in 1991, biological oxygen demand was nearly halved (90th percentile from 8.1 to 4.6 mg/L), ammonia peaks dropped more than 7‐fold (90th percentile from 3.9 to 0.53 mg/L), whereas dissolved oxygen climbed consistently above 60% saturation (10th percentile from 49 to 64%) at a sampling point 2 km downstream of the wastewater treatment plant. A sustained increase in the number of macroinvertebrate species was evident from that point. River flow did not change, temperature rose slightly, and the major metal concentrations declined steadily over most of the monitoring period. Neither the introduction of phosphate stripping in 1999 nor the use of tertiary granular activated charcoal from 2008 to 2014 had strong positive effects on subsequent macroinvertebrate diversity. That the diversity still had not reached the ideal status by 2016 may be related to the modest habitat quality, agricultural pesticides, and limited recolonization potential in the catchment. The results indicate that urban wastewaters, with their chemical pollutants, are today probably not the biggest threat to the macroinvertebrate diversity of multiply stressed lowland rivers in the United Kingdom. *Environ Toxicol Chem* 2019;38:1820–1832. © 2019 The Authors. *Environmental Toxicology and Chemistry* published by Wiley Periodicals, Inc. on behalf of SETAC

## Graphical abstract

Transformation in the fortunes of a macroinvertebrate population in a wastewater dominated river. ASPT = average score per taxon; LOQ = limit of quantification.

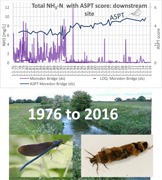

## INTRODUCTION

From the end of World War II most wastewater from inland towns and cities in developed countries has been dealt with by secondary biological treatment, and the era of lifeless and putrefying rivers is behind us (Hynes 1960; Johnson and Sumpter 2014). In Europe, a further stimulus came from the introduction of the Urban Waste Water Directive in 1991 (UWWD; Council Directive 91/271/EEC), where advanced treatment was required for sensitive waters (generally leading to activated sludge replacing trickling filter in towns with a population more than 10 000). However, our appetite to use more and more chemicals in both industry as well as our homes has grown and continues to grow at an astonishing rate. The range of chemicals found to escape in wastewater is extraordinary (Richardson and Ternes 2014). Consequently, a concern exists that many of these chemicals from human activities are harming wildlife today (Malaj et al. 2014).

Historically, studying macroinvertebrate assemblages and their abundances in rivers has been a powerful tool in establishing the extent of pollution harming wildlife. Back in 1902 it was noted that the range of different organisms present could be predicted depending on the degree of decaying organic matter present and vice versa, which gave rise to the “saprobic index” (Kolwitz and Marsson 1902; Sladecek and Tucek 1975). Further developments in the study of different macroinvertebrate taxa gave these each a score from 1 to 10 based on observations of their apparent organic pollution tolerance. Thus, the Biological Monitoring Working Party (BMWP) score is the sum of adding the scores of all the taxa found at a site, whereas the average score per taxon (ASPT) is a similar value showing how attractive the site is to sensitive organisms (Armitage et al. 1983). A theoretical understanding of life traits that might make a macroinvertebrate more vulnerable to pollution impacts (such as those taxa sensitive to organic toxicants, not readily able to recolonize or only reproducing once per year) has led to other indices such as the “Species at Risk” (SPEAR) index for pesticides (Liess and von der Ohe 2005) and more recently for habitat degradation (von der Ohe and Goedkoop 2013). A different approach coming from the chemical side is that by knowing the concentration of a range of hazardous chemicals present and hence the multisubstance potentially affected fraction of species likely to be harmed by these chemicals at those concentrations, one could predict the extent of macroinvertebrate diversity depletion (Posthuma and de Zwart 2012; Posthuma et al. 2016).

So what can a return to studying macroinvertebrate diversity tell us about damage being inflicted by the modern cocktail of chemicals, particularly the personal care products and pharmaceuticals (PCPPs) of today? Spot sampling has often revealed a reduced diversity or absence of some sensitive species downstream of wastewater treatment plants (WWTPs; Canobbio et al. 2009; Ginebreda et al. 2010; Bunzel et al. 2013; Stalter et al. 2013; Burdon et al. 2016). Many of these authors were tempted to put this reduced diversity down to the presence of the mixture of chemicals escaping in wastewater, although the picture can be confounded by the introduction of more fine sediments and perhaps fewer macrophytes reducing the downstream habitat quality (Piliere et al. 2014). If the micro‐organic chemicals present in wastewater are the problem, then it might be assumed that taking away the wastewater effluent entirely or using an advanced tertiary treatment process would lead to recovery of the macroinvertebrate diversity. In a study of the small Vistre River in France and the White River in the United States, closing a poorly functioning WWTP or replacing it with a dramatically improved process did indeed lead to the return of some sensitive taxa within the limits of a degraded habitat (Crawford et al. 1992; Arce et al. 2014). A potentially more valuable study to test the micro‐organic contaminant hypothesis was that of the impact of introducing tertiary ozonation to eliminate all organic contaminants from a WWTP in Switzerland which had an existing acceptably functioning biological treatment stage (Hollender et al. 2009; Ashauer 2016). Unfortunately, although that study did appear to show a benefit as measured by the SPEAR index (curiously the community structure downstream of the WWTP becoming better than that upstream), the trial was only run for 1 yr. Almost all the studies discussed share the same weakness, which is that they represent the result from only a limited period of monitoring. The problems in assessing the results of short‐term studies include that the rate of recolonization depends on the local circumstances and there can be surprisingly high natural variations in the presence or abundance of different taxa from year to year. Many of these studies fail to report the reference condition (ideal diversity) to provide context. More importantly, these previous macroinvertebrate wastewater studies do not tell us whether things are getting worse or better over time. In the United Kingdom a method called the River Invertebrate Prediction and Classification System (RIVPACS) is used to put observed macroinvertebrate communities into context. This compares the observed macroinvertebrate community at any geographic location to that at an unimpacted site of similar elevation and geology (Wright et al. 1993).

Thus, questions remain over whether the current mixture of chemicals which escape in wastewater are harming wildlife, including whether things are getting better or worse and whether stringent tertiary treatment would provide notable benefits. In 2008 the United Kingdom supported the National Demonstration Project, which included measuring the removal effectiveness of some tertiary wastewater treatments. A major component was the installation of granular activated charcoal (GAC) at the Swindon WWTP on the River Ray (a 12‐km‐long tributary of the River Thames) which treated all the effluent. This tertiary treatment ran for a trial period from 2008 until the end of 2014. This large WWTP at Swindon dominates the River Ray and contributes up to 80% of the flow in midsummer (Balaam et al. 2010). A project called Endocrine Disruption in Catchments (EDCAT) looked at the removal of steroid estrogens before and after GAC installation at Swindon and the condition of local stickleback fish (*Gasterosteus aculeatus* L. 1758; Grover et al. 2011; Pottinger et al. 2011a, 2011b). No other deliberate biological monitoring was carried out to coincide with the implementation of GAC treatment at that time. Fortunately, this river has had consistent chemical and macroinvertebrate monitoring starting from the late 1970s to the present day, carried out by the Environment Agency and its predecessors.

The approach of the present study was to review the historic routine Environment Agency monitoring data, going back to the early 1970s, on macroinvertebrates together with chemical determinands, flow, and temperature along the river to understand the impact of changes in wastewater treatment at Swindon. The aim was to establish whether key changes in river water quality, following improvements in Swindon wastewater treatment, could be reconciled with changes in the indigenous macroinvertebrate community. Thus, the present study attempted to test the following hypotheses: 1) improved biological treatment associated with a switch from trickling filter to nitrifying activated sludge will have a detectable beneficial impact on local macroinvertebrate diversity and abundance; 2) phosphate (P) stripping (which started in 1999) will have a detectable beneficial impact on local macroinvertebrate diversity and abundance; 3) GAC tertiary treatment (eliminating micro‐organic contaminants) will have a detectable beneficial impact on local macroinvertebrate diversity and abundance.

## METHODOLOGY

### Description of the site

In 1821 Swindon was described as a quiet market town with a population of 12 455, but this changed with the establishment of the Great Western Railway and the decision to locate all company manufacturing and repair facilities to the town in the early 1840s. The town's industrial development continued into the modern era with the addition of steelwork and car manufacturing. Between 1861 and 2011 the population of the Swindon urban area grew more than 10‐fold, whereas the population of Britain as a whole grew less than 3‐fold in the same period (GB Historical GIS and University of Portsmouth, 2019a,b). By the late 19th century, wastewater treatment for this growing town had become necessary; and a map from 1900 (Ordnance Survey County series Wiltshire sheet XV.3. second edition 1900 25 inch to the mile [ca. 1:2500]) shows rectangular trickling filter beds and sludge pits. Different combinations of these continued to be used until an upgrade to activated sludge treatment occurred in 1991, by which time the Swindon urban area population had reached over 170 800 people (GB Historical GIS and University of Portsmouth, 2019a). The most recent population estimate, for 2017, is >220 300 (Swindon Borough Council 2018).

The introduction of the UWWD and the ability of the newly privatized water companies (privatization occurred in 1989) to borrow money are considered to have speeded up improvements during this period (H. Brett, Thames Water, Reading, UK, personal communication). Between 1990 and 2015 £26 billion was spent on sewage (wastewater) treatment improvements across England (Department for Environment, Food and Rural Affairs 2012).

In 1999, a phosphate (PO_4_) stripping process began operating at the Swindon WWTP because reduction of nutrient discharge was another requirement of the UWWD. In 2008 as part of an agreement between the Department for Environment, Food and Rural Affairs, the Office of Water Services and the Water Industry, Thames Water, agreed to install a GAC unit at Swindon WWTP to establish its efficiency at removing endocrine‐disrupting chemicals. Table 1 summarizes the major changes in the treatment process at Swindon.

**Table 1:** History of major changes made at Swindon wastewater treatment plant

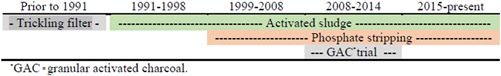				

**Figure 1:**
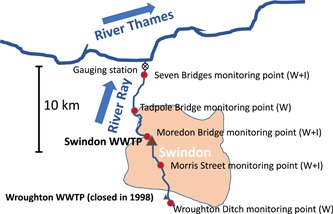
Map of the River Ray with the Environment Agency water sampling and invertebrate monitoring sites as well as the approximate extent of the Swindon urban area. I = invertebrate monitoring site; W = water sampling site; WWTP = wastewater treatment plant.

Upstream of Swindon a small WWTP at Wroughton operated until 1998; from 1991 this had a consented dry weather flow of 2000 m^3^/d or less than one‐twentieth of the 44 300 m^3^/d that Swindon WWTP was allowed (Lewis et al. 1989). Thus, the available upstream macroinvertebrate monitoring site at Morris Street was also receiving some effluent from Wroughton WWTP until 1998 (Figure 1), besides being influenced by the agricultural land use upstream of Swindon. Based on Google Earth estimates, made by the authors in April 2019, upstream of the large Swindon WWTP the city occupies an area of approximately 4407 ha, whereas farther upstream the small rural area leading to Wroughton village and its old WWTP comprises only 200 ha.

**Figure 2:**
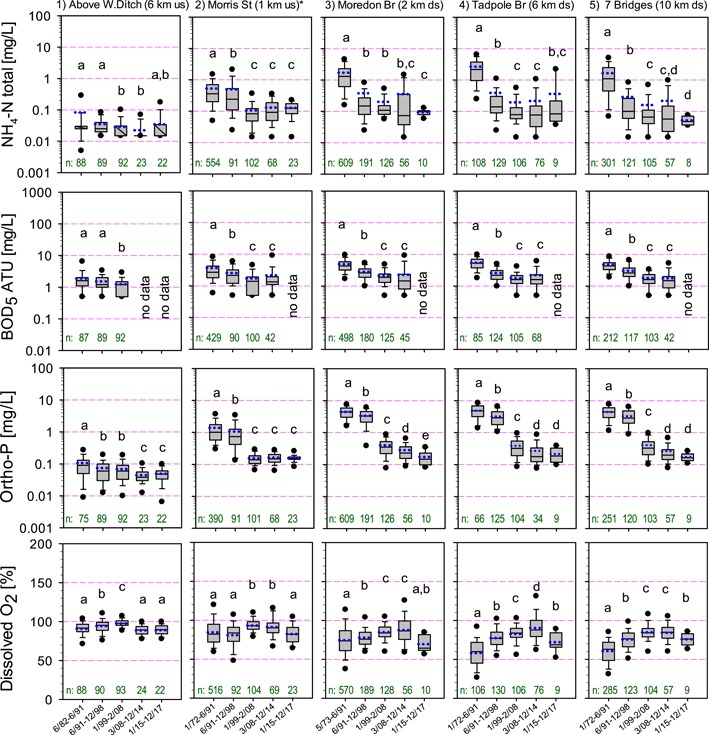
Summary of major water quality determinands over time from upstream to downstream of Swindon WWTP (us: upstream, ds: downstream). Ammoniacal nitrogen as N (total NH4‐N), biological oxygen demand (BOD5‐ATU), phosphate and dissolved oxygen in river water. The time blocks shown coincide with the major changes at Swindon WWTP. The boxes show 25‐, 50‐ and 75‐percentiles, with 10‐ and 90‐percentile whiskers, 5‐ and 95‐ percentiles as symbols and short dotted lines denoting the means. Values <LOQ were entered as ½ LOQ. Different letters denote that the time groups are significantly different at α = 5% (ANOVA and Student s *T*‐test for equal or unequal variances according to *F*‐test, log‐transformed data except for % dissolved oxygen). The sample size n is also given for each group. *Until 1998 the small Wroughton WWTP discharged upstream of Morris Street.

### The river and its monitoring data

The River Ray is 12 km long from the Swindon wastewater discharge point to the confluence with the River Thames (Figure 1). Above Swindon only small streams or brooks exist, with the mean annual flow increasing 5‐fold with the arrival of treated effluent in the river (80% of flow being treated effluent). Such negligible wastewater dilution is unusual for Europe but not unknown, for example, the 5th percentile annual median dilution factor for the UK river network is slightly over 2; only Spain is worse in Europe for this statistic (Keller et al. 2014). At the confluence with the Thames, the mean annual flow of the River Ray still comprises 65% effluent (Balaam et al. 2010).

The following information was available and suitable for the present study (for sources, see *Data Accessibility* section). We used 3 major macroinvertebrate monitoring sites with at least 36 records each, dating back in some cases to 1977: 1 km upstream at Morris Street and 2 km and 10 km downstream of the Swindon WWTP at Moredon Bridge and Seven Bridges (Figure 1). The remaining 2 monitoring sites in Figure 1 were mainly for water samples and have only been monitored for macroinvertebrates occasionally. Detailed community information (i.e., presence and abundance of taxa) was available and provided by the Environment Agency. Typically, 2 macroinvertebrate surveys were carried out per year. To examine this data set, abundances were log10 (*x* + 1)–transformed; and all taxa were grouped by family for the BMWP and ASPT indices, whereas the 2 SPEAR indices were preferably applied to the species level. The BMWP is based on indicator values between 1 (tolerant) and 10 (sensitive), which are added to derive the final score (Armitage et al. 1983). The SPEAR indices again are based on the relative share of sensitive species in a community. For reference conditions, the BMWP and ASPT values are compared to the appropriate unimpacted site (RIVPACS method; Wright et al. 1993), whereas for the SPEAR index, a share of approximately 50% sensitive individuals is to be expected under undisturbed conditions according to pure chance (von der Ohe et al. 2007); that is, if there is no pressure, species might as well be tolerant as sensitive.

At the same locations as the macroinvertebrate monitoring and at Tadpole Bridge, detailed water quality analysis has been carried out since 1972 or 1973; and a slightly shorter time series (since 1982) is available at the far upstream monitoring point at Wroughton Ditch. This included temperature, ammonium, oxidized nitrogen forms, dissolved oxygen, biological oxygen demand (BOD), phosphate, and a range of hazardous chemicals. These were typically monthly samples, though the number of samples per year and site could vary from only 2 to over 50. Regarding water temperature, to avoid a bias for years where the sampling frequency differed between seasons, a 2‐step approach was adopted for calculating the annual average water temperature. In this case, an average value was first calculated per season (winter, December of the previous year to February; spring, March to May; summer, June to August; autumn, September to November), and then the annual average was calculated as the average of the 4 seasons; years without a value in one or more seasons were omitted. The statistical and other calculations were carried out in Excel 2013 with Data Analysis Add‐in.

Two river habitat surveys were carried out, one in 1994 within Swindon and one in 2007 at a site farther downstream between Moredon Bridge and Seven Bridges. These surveys examined the condition of the riverbed and banks together with the range of macrophytes present.

## RESULTS AND DISCUSSION

### Summary of changes to the River Ray water quality over time

For the major water quality determinands of BOD, dissolved oxygen, and ammonia/ammonium combined at the downstream sites, an improvement can be seen from the 1991 time period onward (Figure 2; Supplemental Data, Figure S1), whereas the most dramatic improvement for PO_4_ begins in 1999. These changes coincide with the major Swindon WWTP upgrades (Table 1). Conditions remained relatively good at the most upstream site of all at Wroughton Ditch (6 km upstream), but at the other site upstream of Swindon WWTP, Morris Street (1 km upstream), conditions only improved after the closure of the small WWTP at Wroughton in 1998.

Thus, at the Moredon Bridge monitoring site, 2 km downstream, comparing the pre‐1991 period with that after the arrival of activated sludge but before PO_4_ stripping (i.e. 1991 to 1998), BOD was nearly halved (5‐d BOD 90th percentile reduced from 8.1 to 4.6 mg/L, average 4.9 to 2.8 mg/L), the ammonium (and consequently ammonia) peaks dropped more than 7‐fold (total ammoniacal nitrogen 90th percentile reduced from 3.9 to 0.53 mg/L), and dissolved oxygen remained predominantly above 60% saturation (dissolved oxygen 10th percentile increased from 49 to 64%). During the tertiary GAC plant trial from 2008 to 2014, we could not observe any consistent impact on any of the measured chemicals in the River Ray (Figure 2 and data not shown). Unfortunately, PCPPs are not routinely measured by the Environment Agency. In a previous study, estrogenic and antiandrogenic chemicals were measured in water samples from the River Ray (Grover et al. 2011). These showed overall lower values for 2008 when the GAC unit was in operation compared with 2006 and 2007 when it was not. In laboratory‐scale experiments, GAC has been shown to reduce the concentrations of endocrine disrupters, such as estrogenic chemicals, significantly (Fuerhacker et al. 2001; Zhang and Zhou 2005; Snyder et al. 2007; Liu et al. 2009).

With the exception of iron, all the metals for which sufficient data were available showed declining concentrations both in the WWTP effluent and in the river downstream throughout the monitoring period, as shown for the example of cadmium in Supplemental Data, Figure S2. Iron did not follow this trend because it was introduced as FeCl_3_ to the Swindon WWTP as a phosphorus stripping treatment from 1999. There was some change in iron concentrations in the WWTP effluent when GAC was introduced (Supplemental Data, Figure S3), but iron dosing had no noticeable effect on the concentrations downstream in the river (Supplemental Data, Figure S4). The other metals roughly followed exponential decay curves with “half‐lives” between approximately 3 yr and 2 decades (Table 2). These apparent half‐lives are likely to be mainly the result of reduced industrial and domestic consumption and discharge of metals over time, although perhaps improved removal of suspended solids in wastewater treatment played a role. In each case, the decline in river metal concentrations followed that in the WWTP effluent.

**Table 2:** Decline in metal concentrations over time reported as “half‐lives” calculated from quantifiable concentrations^a^

Metal	Site	Measurement period	*n* all data	% <LOQ^b^ all data	*t*_1/2_ (yr)	*r* ^2^	Concentration 6/89–7/90 average (SD; µg/L)^c^	*n* (% <LOQ) 6/89–7/90	Comments
Boron	WWTP effluent	1/90–10/08	91	2.2%	6.3	0.72	847 (584)	3 (0%)	
	7 Br (10 km ds)	1/88–07/08	234	2.6%	8.4	0.47	903 (388)	20 (0%)	
Cadmium	WWTP effluent	1/73–11/16	360	53.6%	3.5	0.74	0.22 (0.19)	34 (12%)	>90% <LOQ after 1990, *t*_½_ for 1973–1990 = 2.7 yr (*r* ^2^ = 0.84)
	T. Br (6 km ds)	3/77–04/15	396	52.3%	5.7	0.77	No data		
	7 Br (10 km ds)	7/74–01/15	399	37.8%	6.0	0.75	0.32 (0.28)	20 (15%)	
Chromium	WWTP effluent	1/73–10/08	327	77.4%	9.0	0.62	2.6 (0.4)	32 (97%)	By 1991 values were rarely above the LOQ of 5 µg/L
	7 Br (10 km ds)	8/73–7/08	373	64.3%	7.6	0.49	3.9 (3.4)	21 (90%)	
Copper	WWTP effluent	1/88–10/08	137	3.6%	8.7	0.66	14 (3.9)	32 (0%)	Values before 1988 were omitted because resolution was only to 10 µg/L
	7 Br (10 km ds)	7/88–7/08	253	5.1%	13.1	0.38	11 (5.0)	23 (4%)	
Copper dissolved	T. Br (6 km ds)	5/89–12/13	290	9.0%	10.7	0.68	6.8 (4.0)	21 (38%)	
	7 Br (10 km ds)	4/86–9/17	345	19.4%	12.2	0.54	7.8 (5.2)	18 (28%)	
Iron	WWTP effluent	8/76–11/17	304	4.9%			120 (40)	3 (0%)	Approximately 5‐fold increase with P stripping from 1/99; during GAC about half the values were low again
	7 Br (10 km ds)	10/82–7/08	223	0%			619 (910)	10 (0%)	No trend detectable, but there are very few data points before P stripping started in 1999
Iron dissolved	7 Br (10 km ds)	1/88–9/17	243	23.0%			No data		No trend detectable, but there are very few data points before P stripping started in 1999
Lead	WWTP effluent	8/76–10/08	161	48.4%	5.4	0.65	2.4 (1.2)	31 (35%)	
Lead dissolved	7 Br (10 km ds)	3/94–09/17	100	21.0%	5.7	0.57	No data		Before 1994 and between 2002 and 2014 detection limit was too high, so those years were omitted
Nickel	WWTP effluent	1/73–10/08	327	27.5%	10.8	0.56	6.3 (3.3)	32 (34%)	Values may have stabilized around the mid‐1980s
	7 Br (10 km ds)	7/74–7/08	372	42.7%	11.4	0.65	5.9 (4.4)	22 (59%)	
Zinc	WWTP effluent	1/73–10/08	327	0.9%	15.8	0.45	36 (12)	32 (0%)	
	7 Br (10 km ds)	7/74–09/17	491	1.6%	22.0	0.39	35 (9.5)	23 (0%)	

^a^ Data are from the sites with the most comprehensive records in the river. The average concentrations measured in the 2 yr prior to the WWTP upgrade to activated sludge treatment are also given.

^b^ Percentage of measurements less than the limit of quantification. These were ignored for the regression calculation.

^c^ For the average calculation <LOQ was entered as one‐half the limit of quantification.

ds = downstream; 7 Br = Seven Bridges; GAC = granular activated charcoal; LOQ = limit of quantification; T. Br = Tadpole Bridge.

To assess the risks posed by individual hazardous chemicals, a risk‐ranking exercise was carried out. The hazardous chemicals monitored in the River Ray by the Environment Agency with measurements routinely above the level of quantification were Cu, Zn, NH_3_, Ni, Al, Mn, Pb, Fe, di‐2‐ethylhexyl phthalate, Cd, benzo[a]pyrene, As, Hg, and Cr. Using the risk‐ranking method of comparing the highest river measurements (90th percentile) with the lowest most sensitive ecotoxicity values (10th percentile), we did not find risks exceeding the 1.0 ratio for any of these chemicals (Johnson et al. 2017). Although Cu and Zn showed the highest relative risk, they did not exceed the United Kingdom environmental quality standard for hard water (>250 µg/L total hardness as CaCO_3_, 97.5% of all hardness measurements were in this range) of 28 µg/L Cu and 125 µg/L Zn except for Cu in 4 of 345 measurements at Seven Bridges. However, before 1991 both ammonia and dissolved oxygen (Supplemental Data, Figure S1) had poor episodes which could be classified as failing the environmental quality standard now used in the Water Framework Directive with, for example, oxygen saturation going sometimes below 20% in the late 1980s. Note that all samples were taken in daytime when photosynthesis would have increased dissolved oxygen levels compared to the night.

### Summary of changes to the River Ray water flow and temperature over time

**Figure 3:**
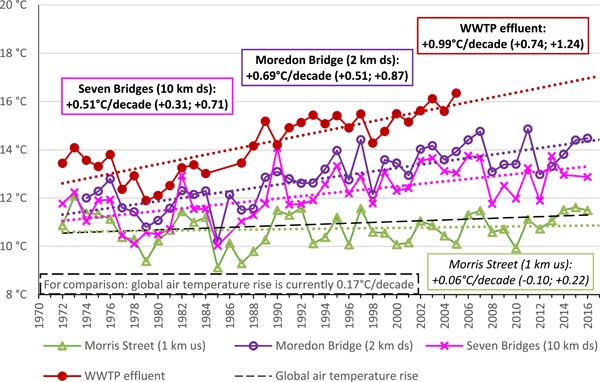
Annual average water temperature of the River Ray upstream (us) and downstream (ds) of the Swindon wastewater treatment plant (WWTP), together with the temperature of the WWTP effluent itself. The slopes of the linear regressions together with their 95% confidence intervals are also given.

As might be expected with a wastewater‐dominated river, no net change in flows or patterns in the River Ray occurred from the 1970s to the present day (Supplemental Data, Figure S5). However, an increase in average river water temperature over all the years could be seen in the WWTP effluent and at all sites downstream of the WWTP (Figure 3). This temperature increase is approximately 1.0 °C/decade in the WWTP effluent reducing to 0.7 and 0.5 °C/decade in the river 2 and 10 km downstream of the WWTP. The effect was especially strong in winter (December–February), when the increase was 1.3 °C/decade in the effluent and 0.9 °C/decade 2 km downstream of the WWTP. Only a small proportion of this water temperature increase may be explained by global warming, which is currently estimated to increase global air temperatures by 0.17 °C/decade (Christy et al. 2018). No evidence for increasing temperatures was found at the upstream sampling site at Morris Street, confirming that a trend of warming wastewater was the cause.

### River habitat survey

River habitat survey data are limited to 2 sites in this catchment, providing a habitat modification score (a measure of the extent of artificial channel, bank, and riparian characteristics at the site) and the habitat quality assessment (a measure of the extent of natural characteristics at the site). A survey was carried out in 1994 (river habitat survey reference 1142, SU1320085000) in the urban part of Swindon just upstream of the WWTP. Results were typical of an urban area with a habitat modification score of 3465 (class 5, “severely modified”) indicative of an artificial and resectioned channel with artificial banks and limited riparian habitat. The river habitat survey yielded a habitat quality assessment of 33, consistent with the habitat modification score, because it also indicated that there were few natural habitat features. This site is likely to be representative of the urban part of the River Ray and indicates that benthic faunal diversity and abundance could be significantly constrained by poor habitat quality. The second river habitat survey (reference 32753) was done in 2007 and was located approximately 5 km downstream of the urban area (SU1121889010), between Moredon Bridge and Seven Bridges. This survey indicated less modification of the channel, banks, and riparian zone than upstream (habitat modification score = 250; class 3, “obviously modified”); however, the habitat quality assessment was still low (38). Survey details indicated that this later site had more diverse natural vegetation on the banks but that the channel substrate was poorer than at the urban site. This site was deemed roughly representative of the Ray downstream of Swindon, and there too habitat characteristics may limit benthic diversity.

### Macroinvertebrate diversity and ecology scores

To provide an assessment of the diversity of the many different families of macroinvertebrates at a site, the BMWP score is used in many countries in Europe and elsewhere to reveal the impact of a wide range of pressures, especially including organic pollution. Changes in BMWP score are driven by the presence or absence of certain macroinvertebrate families, known to be sensitive or insensitive to high organic loads and related oxygen depletion (Armitage et al. 1983). Likewise, the SPEAR_organic_ and SPEAR_habitat_ indices represent the share of individuals in the macroinvertebrate community that are sensitive to organic toxicants or habitat loss, respectively (von der Ohe and Liess 2004; von der Ohe and Goedkoop 2013). Examples of the different scoring systems of BMWP/ASPT and SPEAR are shown in Table 3.

**Table 3:** Characteristics of some example macroinvertebrate families used in biological indicator systems and their respective stressor indication

Family	BMWP and ASPT	SPEAR_organic_	SPEAR_habitat_	Comment/conclusion
Asellidae	3	0	0	Tolerant to pollution + ubiquitous
Calopterygonidae	8	0	1	Sensitive to oxygen depletion and habitat
Hydrophsycidae	5	0	1	
Tipulidae	5	0	1	
Ceratopogonidae	0	0	1	Tolerant to pollution + sensitive to habitat
Limnephilidae	7	1	1	Sensitive to all stressors
Leptoceridae	10	1	1	

ASPT = average score per taxon; BMWP = Biological Monitoring Working Party; SPEAR = Species at Risk index.

**Figure 4:**
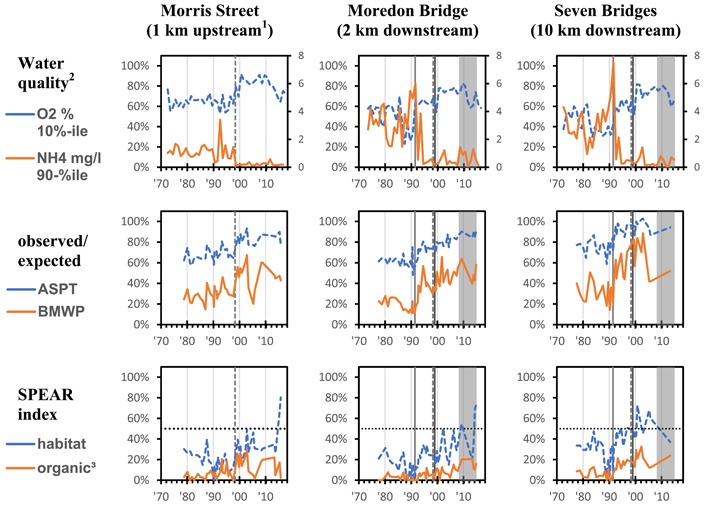
Dissolved oxygen (percent saturation) and ammonium concentrations at 3 monitoring sites over time: Morris Street (1 km upstream of Swindon but downstream of the small Wroughton wastewater treatment plant [WWTP]) and Moredon Bridge (2 km) and Seven Bridges (10 km) downstream of the Swindon WWTP compared to 4 biological metrics. Average score per taxon and the Biological Monitoring Working Party are plotted as a percentage of the predicted ideal scores for these sites (https://www.ceh.ac.uk/services/rivpacs-reference-database). For the SPEAR indices 50% is seen as a reference value where sensitive and nonsensitive species are equally likely to occur. This is indicated by a dotted horizontal line. The upgrade of Swindon WWTP to activated sludge in 1991 and the introduction of P‐stripping in 1999 are marked with solid vertical lines. The gray shading denotes the period from 2008‒2014 when granular activated charcoal was used and the dashed vertical line denotes the closure of Wroughton WWTP in 1998. ^1^Until 1998 there was a small WWTP upstream of this site. ^2^Excluding 0 to 3 yr with fewer than 4 data points. ^3^Excluding *Asellus*.

As an example, the trends in the BMWP, ASPT, and SPEAR indices over time have been compared to ammonium concentrations at Morris Street, Moredon Bridge, and Seven Bridges (Figure 4). Ammonium (linked to ammonia) is one of the big 3 water quality characteristics which dramatically improved in 1991 following the upgrade to the activated sludge process at Swindon WWTP (Figure 2). The trend in these aggregated scores appears to show a dramatic and then sustained improvement for macroinvertebrates sensitive to organic pollution (i.e., the BMWP indicator value almost tripled) after 1991 at Moredon Bridge and Seven Bridges, which are downstream of Swindon. It is important to recall that the Moredon Bridge site is only 2 km downstream of Swindon WWTP, where the flow is 80% wastewater effluent on average. The improving trends in SPEAR_habitat_ and SPEAR_organic_ at this site were less pronounced and at a lower level compared to the BMWP and ASPT indices, but this improvement also started after 1991 (Figure 4). The indices at Morris Street (1 km upstream) were generally higher but still at a far from unimpacted level, with SPEAR_organic_ values being again the lowest. These indices combined appear to show an improving habitat quality downstream but with some limiting factor(s) still present. However, the very low levels of SPEAR_organic_ suggesting a rather low recovery of taxa sensitive to organic toxicants may indicate residual agricultural pressures (including pesticides) as well as a weak or missing upstream recolonization potential from Morris Street (von der Ohe and Goedkoop 2013). Later improvements seen at Morris Street could be linked to the closure of the upstream Wroughton WWTP in 1998.

Generally, the macroinvertebrate community at Seven Bridges, which is located 10 km farther downstream of Swindon WWTP, had higher index values compared to the site at Moredon Bridge (2 km downstream of Swindon WWTP). This may be attributable to greater dilution of the Swindon wastewater as well as a generally better habitat, as reflected in the river habitat survey. The latter might also be reflected in the relatively higher SPEAR_habitat_ values compared to the similarly low SPEAR_organic_ values at that site, when compared with the communities closer to Swindon WWTP at Moredon Bridge.

The 2008 to 2014 period of GAC tertiary treatment does not stand out as having a significant impact on the macroinvertebrate trends, although the SPEAR_organic_ indicator increases in this period (Figure 4).

### Changes in macroinvertebrates at the family level at Moredon Bridge

We can zoom in from this broad macroinvertebrate scoring assessment to look at how the individual families have fluctuated at the effluent‐dominated Moredon Bridge site from the 1970s to the present day. The Asellidae (principally represented by *Asellus aquaticu*s L. 1758), a family known to be abundant in organic enriched contexts (Maltby 1991) and able to tolerate chemical pollution (von der Ohe et al. 2007), have had a constant presence; but their abundance was greatest in the pre‐1991 poor water quality period (Supplemental Data, Figure S6).

There were several families whose presence and abundance seemed to be rather independent of water quality change (Supplemental Data, Figure S10). These families were Baetidae and Leptophlebiidae (Ephemeroptera); Chironomidae and Psychodidae (Diptera); Dytiscidae, Haliplidae, and Hydrophilidae (Coleoptera); Lymaeidae, Physidae, and Planorbidae (Gastropoda); Gammaridae (Amphipoda); Glossiphoniidae (Hirudinea); and Psychomiidae (Trichoptera). These families have low scores on the BMWP scale (see some examples in Table 3) and are classified as tolerant for SPEAR_organic_.

**Figure 5:**
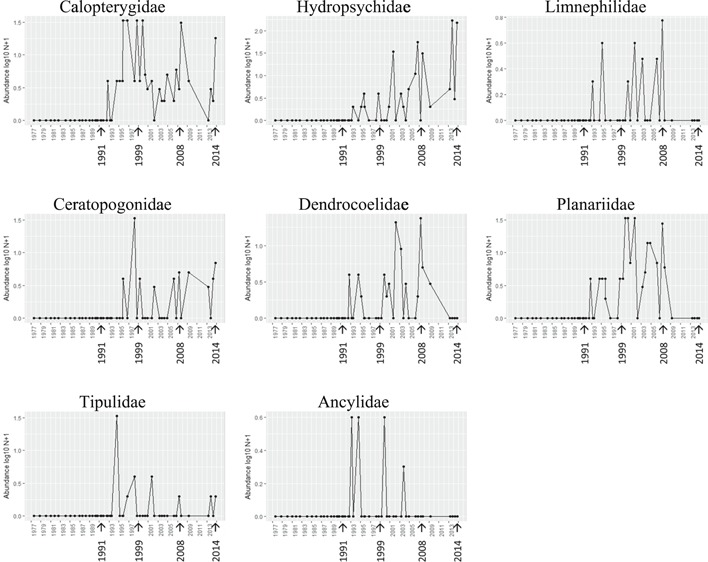
Families which arrived post‐1991 at Moredon Bridge 2 km downstream of Swindon wastewater treatment plant, in association with improved water quality (arrows indicate 1991 change to activated sludge, 1999 introduction of PO_4_ stripping, 2008–2014 granular activated charcoal trial).

**Figure 6:**
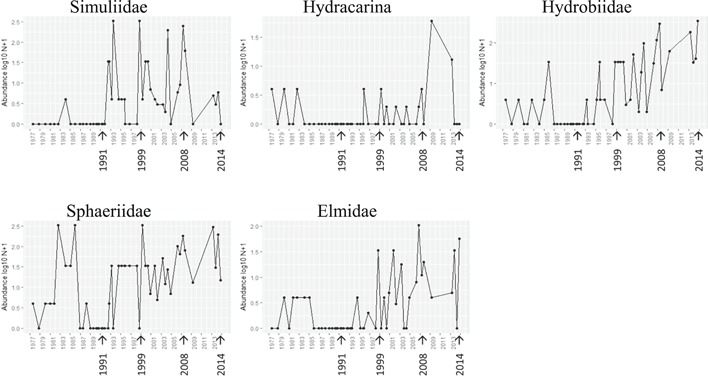
Families which returned to Moredon Bridge, 2 km downstream of Swindon wastewater treatment plant, post‐1991 following the particularly poor water quality period of the late 1980s (arrows indicate 1991 change to activated sludge, 1999 introduction of PO_4_ stripping, 2008–2014 granular activated charcoal trial).

**Figure 7:**
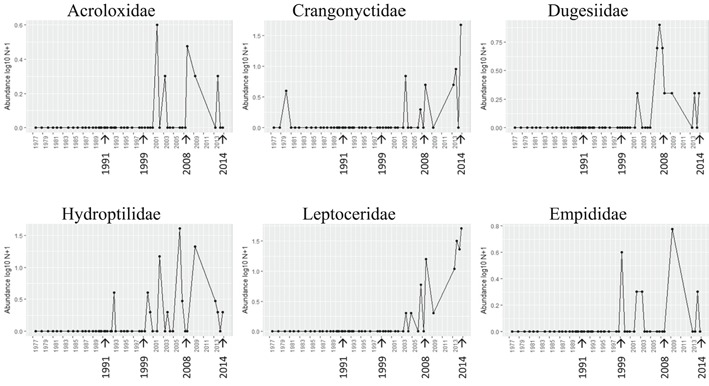
Families which arrived or increased at Moredon Bridge, 2 km downstream of Swindon wastewater treatment plant, later in the post‐2000 period, possibly linked to further improving water quality, such as PO_4_ stripping from 1999.

Some families were only recorded at the Moredon Bridge site post‐1991 (Figure 5; Supplemental Data, Figure S7); of these, some are associated with cleaner water and lower organic loading (particularly Calopterygidae, BMWP value 8/10), although others are deemed to not be sensitive to organic pollution (e.g., Ceratopogonidae; Table 3). The families in Figure 6 and Supplemental Data, Figure S8, had a transitory disappearance during the period of worst water quality (particularly low dissolved oxygen levels in the late 1980s; see Figure 4; Supplemental Data, Figure S1). These included the Hydracarina, for which water quality requirements are not well known, whereas the other families have modest BMWP values, between 3 and 5. New families continued to slowly appear in the post‐2000 period (Figure 7; Supplemental Data, Figure S9), which may reflect continuing improved water quality. Of these, 3 taxa may only appear to increase over time as a result of recording artifacts under the initial BMWP system because Acroloxidae, Crangonyctidae, and Dugesiidae may have earlier been recorded with other families (Ancylidae, Gammaridae, and Planariidae, respectively). However, the Hydroptilidae (BMWP score 6) and Leptoceridae (BMWP score 10) families are strongly associated with low organic pollution, and their return would indicate that such conditions were improving.

## CONCLUSIONS

Returning to the original hypotheses, 1) improved biological treatment associated with a switch from trickling filter to nitrifying activated sludge will have a detectable beneficial impact on local macroinvertebrate diversity and abundance is supported; 2) phosphate stripping will have a detectable beneficial impact on local macroinvertebrate diversity and abundance is ambiguous; and 3) GAC tertiary treatment will have a detectable beneficial impact on local macroinvertebrate diversity and abundance is not supported.

The observed improvements in macroinvertebrate diversity and abundance (reflected in all of the macroinvertebrate indices but especially in BMWP) are consistent with a notable improvement in oxygen levels and a strong reduction in BOD and ammonia following the introduction of activated sludge in 1991. Of the other possible drivers of this change, it is unlikely that dramatic improvements in local habitat or reductions in pesticide use occurred over the period 1980 to 2014. There were no big changes in river flow characteristics either (Supplemental Data, Figure S5). The original levels for metals did not exceed environmental quality standards, and there were no notable changes in concentration over the 1990 to 1992 period when the macroinvertebrate diversity suddenly improved. Although warming temperatures might generally enhance macroinvertebrate abundance, this factor probably did not make the critical difference seen between 1990 and 1991. There remains a possibility that an unknown chemical, not measured by the Environment Agency, escaping from the WWTP when it was a trickling filter or from farther upstream, had been suppressing macroinvertebrates before 1991. For some pharmaceuticals, removal performance from trickling filter WWTP can be less than that from activated sludge (Gardner et al. 2013). However, a recent review of chemicals of concern in United Kingdom rivers has not found any chemical to be of higher risk than the metals (Johnson et al. 2017).

The implications of observations in the River Ray are that once the lowest 10th percentile dissolved oxygen does not decline below 60%, the BOD highest 90th percentile does not rise above 5 mg/L, the NH_3_ highest 90th percentile does not rise above 0.6 mg/L (all associated with the Water Framework Directive good status class), and metals or pesticides are not at a toxic level, a consistent and steady improvement in macroinvertebrate diversity can be expected within the limits of the local habitat and the recolonization potential of the catchment.

There was no dramatic distinguishing change in overall macroinvertebrate diversity following PO_4_ stripping, although some new families did arrive post‐1999 and pre‐2008. Over a 6‐yr period, the operation of the GAC unit did not much alter this general improvement in macroinvertebrate diversity, with some families, such as Hydracarina and Leptoceridae, doing well but others, such as Limnephilidae and Planariidae, disappearing again (Figures 5, 6, 7), although there were only limited data for the period post introduction of the GAC unit. The expected dramatic improvement in SPEAR_organic_ was not observed, suggesting that the chemicals escaping the WWTP are not the main drivers of community structure at Moredon Bridge.

Many of the WWTPs across the United Kingdom probably improved performance thanks to pressure from the UWWD during the 1990s. If macroinvertebrates are improving in an effluent‐dominated river like the Ray, this may go some way to explaining the general improvement across the United Kingdom for macroinvertebrate diversity in urban areas reported from the 1990s (Vaughan and Ormerod 2012). These results, however, would not support the widespread introduction of stringent tertiary treatment, like GAC, if macroinvertebrate abundance and diversity were our only concerns. In this case, improving habitats as well as reducing the impacts of agricultural land uses might be more likely than further wastewater tertiary treatment to achieve the desired macroinvertebrate community status. Of course, macroinvertebrates are not the only group of aquatic wildlife we want to protect. But so far we have failed to demonstrate that exposure to regular wastewater harms fish populations (Hamilton et al. 2014; Johnson and Chen 2017), although this does not mean that the residual chemicals in wastewater do not influence individual fish health (Jobling et al. 2006; Pottinger et al. 2013).

## Supplemental Data

The Supplemental Data are available on the Wiley Online Library at DOI: 10.1002/etc.4460.

## Disclaimer

The views expressed here are those of the authors alone.

## Supporting information

This article includes online‐only Supplemental Data.

Supporting information.

## Data Availability

All source data are available on application to the Environment Agency at national.enquiries@environment-agency.gov.uk. Summary invertebrate data from 1990 onward are also online at https://data.gov.uk, and recent water chemistry data (from 2000) can be downloaded at http://environment.data.gov.uk/water-quality.

## References

[etc4460-bib-0001] Arce E , Archaimbault V , Mondy CP , Usseglio‐Polatera P . 2014 Recovery dynamics in invertebrate communities following water‐quality improvement: Taxonomy‐ vs trait‐based assessment. Freshw Sci 33:1060–1073.

[etc4460-bib-0002] Armitage PD , Moss D , Wright JF , Furse MT . 1983 The performance of a new biological water quality score system based on macroinvertebrates over a wide range of unpolluted running‐water sites. Water Res 17:333–347.

[etc4460-bib-0003] Ashauer R . 2016 Post‐ozonation in a municipal wastewater treatment plant improves water quality in the receiving stream. Environ Sci Eur 28:1. 27752436 10.1186/s12302-015-0068-z PMC5044949

[etc4460-bib-0004] Balaam JL , Grover D , Johnson AC , Jürgens M , Readman J , Smith AJ , White S , Williams R , Zhou JL . 2010 The use of modelling to predict levels of estrogens in a river catchment: How does modelled data compare with chemical analysis and in vitro yeast assay results? Sci Total Environ 408:4826–4832. 20673965 10.1016/j.scitotenv.2010.07.019

[etc4460-bib-0005] Bunzel K , Kattwinkel M , Liess M . 2013 Effects of organic pollutants from wastewater treatment plants on aquatic invertebrate communities. Water Res 47:597–606. 23174534 10.1016/j.watres.2012.10.031

[etc4460-bib-0006] Burdon FJ , Reyes M , Alder AC , Joss A , Ort C , Rasanen K , Jokela J , Eggen RIL , Stamm C . 2016 Environmental context and magnitude of disturbance influence trait‐mediated community responses to wastewater in streams. Ecol Evol 6:3923–3939. 27516855 10.1002/ece3.2165 PMC4972221

[etc4460-bib-0007] Canobbio S , Mezzanotte V , Sanfilippo U , Benvenuto F . 2009 Effect of multiple stressors on water quality and macroinvertebrate assemblages in an effluent‐dominated stream. Water Air Soil Pollut 198:359–371.

[etc4460-bib-0061] Christy JR , Po‐Chedley S , Mears C 2018 Troposheric temperature [in “State of the Climate in 2017”]. Bull Amer Meteor Soc 99: S16‐S18. DOI: 10.1175/2018BAMSStateoftheClimate.1

[etc4460-bib-0008] Crawford CG , Wangsness DJ , Martin JD . 1992 Recovery of benthic‐invertebrate communities in the White River near Indianapolis, Indiana, USA, following implementation of advanced treatment of municipal waste‐water. Arch Hydrobiol 126:67–84.

[etc4460-bib-0009] Department for Environment, Food and Rural Affairs . 2012. Waste water treatment in the United Kingdom—2012. London, UK.

[etc4460-bib-0010] Fuerhacker M , Dürauer A , Jungbauer A . 2001 Adsorption isotherms of 17β‐estradiol on granular activated carbon (GAC). Chemosphere 44:1573–1579. 11545523 10.1016/s0045-6535(00)00543-9

[etc4460-bib-0011] Gardner M , Jones V , Comber S , Scrimshaw MD , Coello‐Garcia T , Cartmell E , Lester J , Ellor B . 2013 Performance of UK wastewater treatment works with respect to trace contaminants. Sci Total Environ 456:359–369. 23624009 10.1016/j.scitotenv.2013.03.088

[etc4460-bib-0040] GB Historical GIS, University of Portsmouth . 2019a A vision of Britain through time. Swindon, Wiltshire, UK. [cited 2019 June 20]. Available from: http://www.visionofbritain.org.uk/unit/10104178/cube/TOT_POP

[etc4460-bib-0060] GB Historical GIS, University of Portsmouth . 2019b A vision of Britain through time. Great Britain Dep [cited 2019 June 20]. Available from: http://www.visionofbritain.org.uk/unit/10104178/cube/TOT_POP

[etc4460-bib-0012] Ginebreda A , Munoz I , de Alda ML , Brix R , Lopez‐Doval J , Barcelo D . 2010 Environmental risk assessment of pharmaceuticals in rivers: Relationships between hazard indexes and aquatic macroinvertebrate diversity indexes in the Llobregat River (NE Spain). Environ Int 36:153–162. 19931909 10.1016/j.envint.2009.10.003

[etc4460-bib-0013] Grover DP , Balaam J , Pacitto S , Readman JW , White S , Zhou JL . 2011 Endocrine disrupting activities in sewage effluent and river water determined by chemical analysis and in vitro assay in the context of granular activated carbon upgrade. Chemosphere 84:1512–1520. 21546050 10.1016/j.chemosphere.2011.04.032

[etc4460-bib-0014] Hamilton PB , Nicol E , De‐Bastos ESR , Williams RJ , Sumpter JP , Jobling S , Stevens JR , Tyler CR . 2014 Populations of a cyprinid fish are self‐sustaining despite widespread feminization of males. BMC Biol 12:1. 24417977 10.1186/1741-7007-12-1 PMC3922797

[etc4460-bib-0015] Hollender J , Zimmermann SG , Koepke S , Krauss M , McArdell CS , Ort C , Singer H , von Gunten U , Siegrist H . 2009 Elimination of organic micropollutants in a municipal wastewater treatment plant upgraded with a full‐scale post‐ozonation followed by sand filtration. Environ Sci Technol 43:7862–7869. 19921906 10.1021/es9014629

[etc4460-bib-0016] Hynes HBN . 1960 The Biology of Polluted Waters. Liverpool University Press, Liverpool, UK.

[etc4460-bib-0017] Jobling S , Williams R , Johnson A , Taylor A , Gross‐Sorokin M , Nolan M , Tyler CR , van Aerle R , Santos E , Brighty G . 2006 Predicted exposures to steroid estrogens in UK rivers correlate with widespread sexual disruption in wild fish populations. Environ Health Perspect 114:32–39. 16818244 10.1289/ehp.8050 PMC1874167

[etc4460-bib-0018] Johnson AC , Chen YH . 2017 Does exposure to domestic wastewater effluent (including steroid estrogens) harm fish populations in the UK? Sci Total Environ 589:89–96. 28273597 10.1016/j.scitotenv.2017.02.142

[etc4460-bib-0019] Johnson AC , Donnachie RL , Sumpter JP , Jürgens MD , Moeckel C , Pereira MG . 2017 An alternative approach to risk rank chemicals on the threat they pose to the aquatic environment. Sci Total Environ 599–600:1372–1381. 10.1016/j.scitotenv.2017.05.039 28531948

[etc4460-bib-0020] Johnson AC , Sumpter JP . 2014 Putting pharmaceuticals into the wider context of challenges to fish populations in rivers. Philos Trans R Soc Lond B Biol Sci 369:20130581. 25405969 10.1098/rstb.2013.0581 PMC4213592

[etc4460-bib-0021] Keller VDJ , Williams RJ , Lofthouse C , Johnson AC . 2014 Worldwide estimation of river concentrations of any chemical originating from sewage treatment plants using dilution factors. Environ Toxicol Chem 33:447–452. 24375744 10.1002/etc.2441 PMC4253128

[etc4460-bib-0022] Kolwitz R , Marsson M . 1902 Grundsätze für die biologische Beurtheilung des Wassers nach seiner Flora und Fauna. Mitteilungen aus der Prüfungsanstalt für Wasserversorgung und Abwasserbeseitigung 1:33–72.

[etc4460-bib-0023] Lewis V , Willis D , Killingbeck A , Hopkins E . 1989 River Ray, Wiltshire: Fisheries survey 1989 National Rivers Authority, London, UK.

[etc4460-bib-0024] Liess M , von der Ohe PC . 2005 Analyzing effects of pesticides on invertebrate communities in streams. Environ Toxicol Chem 24:954–965. 15839571 10.1897/03-652.1

[etc4460-bib-0025] Liu ZH , Kanjo Y , Mizutani S . 2009 Removal mechanisms for endocrine disrupting compounds (EDCs) in wastewater treatment—Physical means, biodegradation, and chemical advanced oxidation: A review. Sci Total Environ 407:731–748. 18992918 10.1016/j.scitotenv.2008.08.039

[etc4460-bib-0026] Malaj E , von der Ohe PC , Grote M , Kuhne R , Mondy CP , Usseglio‐Polatera P , Brack W , Schafer RB . 2014 Organic chemicals jeopardize the health of freshwater ecosystems on the continental scale. Proc Natl Acad Sci USA 111:9549–9554. 24979762 10.1073/pnas.1321082111 PMC4084479

[etc4460-bib-0027] Maltby L . 1991 Pollution as a probe of life‐history adaptation in *Asellus aquaticus* (Isopoda). Oikos 61:11–18.

[etc4460-bib-0029] Piliere A , Schipper AM , Breure TM , Posthuma L , de Zwart D , Dyer SD , Huijbregts MAJ . 2014 Unraveling the relationships between freshwater invertebrate assemblages and interacting environmental factors. Freshw Sci 33:1148–1158.

[etc4460-bib-0030] Posthuma L , de Zwart D . 2012 Predicted mixture toxic pressure relates to observed fraction of benthic macrofauna species impacted by contaminant mixtures. Environ Toxicol Chem 31:2175–2188. 22729941 10.1002/etc.1923

[etc4460-bib-0031] Posthuma L , Dyer SD , de Zwart D , Kapo K , Holmes CM , Burton GA . 2016 Eco‐epidemiology of aquatic ecosystems: Separating chemicals from multiple stressors. Sci Total Environ 573:1303–1319. 27519323 10.1016/j.scitotenv.2016.06.242

[etc4460-bib-0032] Pottinger TG , Cook A , Jürgens MD , Rhodes G , Katsiadaki I , Balaam JL , Smith AJ , Matthiessen P . 2011a Effects of sewage effluent remediation on body size, somatic RNA:DNA ratio, and markers of chemical exposure in three‐spined sticklebacks. Environ Int 37:158–169. 20851469 10.1016/j.envint.2010.08.012

[etc4460-bib-0033] Pottinger TG , Cook A , Jürgens MD , Sebire M , Henrys PA , Katsiadaki I , Balaam JL , Smith AJ , Matthiessen P . 2011b Indices of stress in three‐spined sticklebacks *Gasterosteus aculeatus* in relation to extreme weather events and exposure to wastewater effluent. J Fish Biol 79:256–279. 21722123 10.1111/j.1095-8649.2011.03013.x

[etc4460-bib-0034] Pottinger TG , Henrys PA , Williams RJ , Matthiessen P . 2013 The stress response of three‐spined sticklebacks is modified in proportion to effluent exposure downstream of wastewater treatment works. Aquat Toxicol 126:382–392. 23021553 10.1016/j.aquatox.2012.09.002

[etc4460-bib-0035] Richardson SD , Ternes TA . 2014 Water analysis: Emerging contaminants and current issues. Anal Chem 86:2813–2848. 24502364 10.1021/ac500508t

[etc4460-bib-0036] Sladecek V , Tucek F . 1975 Relation of saprobic index to BOD_5_ . Water Res 9:791–794.

[etc4460-bib-0037] Snyder SA , Adham S , Redding AM , Cannon FS , DeCarolis J , Oppenheimer J , Wert EC , Yoon Y . 2007 Role of membranes and activated carbon in the removal of endocrine disruptors and pharmaceuticals. Desalination 202:156–181.

[etc4460-bib-0038] Stalter D , Magdeburg A , Quednow K , Botzat A , Oehlmann J . 2013 Do contaminants originating from state‐of‐the‐art treated wastewater impact the ecological quality of surface waters? PLoS One 8:10. 10.1371/journal.pone.0060616 PMC3620539 23593263

[etc4460-bib-0039] Swindon Borough Council . 2018 Swindon's Joint Strategic Needs Assessment. Swindon, Wiltshire, UK. [cited 2019 June 20]. Available from: http://www.swindonjsna.co.uk/dna/population-estimates-projections

[etc4460-bib-0041] Vaughan IP , Ormerod SJ . 2012 Large‐scale, long‐term trends in British river macroinvertebrates. Glob Chang Biol 18:2184–2194.

[etc4460-bib-0042] von der Ohe PC , Goedkoop W . 2013 Distinguishing the effects of habitat degradation and pesticide stress on benthic invertebrates using stressor‐specific metrics. Sci Total Environ 444:480–490. 23291651 10.1016/j.scitotenv.2012.12.001

[etc4460-bib-0043] von der Ohe PC , Liess M . 2004 Relative sensitivity distribution of aquatic invertebrates to organic and metal compounds. Environ Toxicol Chem 23:150–156. 14768879 10.1897/02-577

[etc4460-bib-0044] von der Ohe PC , Prub A , Schafer RB , Liess M , de Deckere E , Brack W . 2007 Water quality indices across Europe—A comparison of the good ecological status of five river basins. J Environ Monit 9:970–978. 17726558 10.1039/b704699p

[etc4460-bib-0046] Wright JF , Furse M , Armitage P . 1993 RIVPACS—A technique for evaluating the biological quality of rivers in the UK. Eur Water Pollut Control 3:15–25.

[etc4460-bib-0047] Zhang Y , Zhou JL . 2005 Removal of estrone and 17β‐estradiol from water by adsorption. Water Res 39:3991–4003. 16126247 10.1016/j.watres.2005.07.019

