# Supplementary material for: What Works? the Influence of Changing Wastewater Treatment Type, Including Tertiary Granular Activated Charcoal, on Downstream Macroinvertebrate Biodiversity Over Time
Source: Environ Toxicol Chem. 2019 Jul 30;38(8):1820–32. doi: 10.1002/etc.4460 (PMC6851886; doi:10.1002/etc.4460)
Supplement: Supplementary file 1 — Supporting information. [file ETC-38-1820-s001.docx]

# Supplementary Information

# What works? The influence of changing wastewater treatment type, including tertiary granular activated charcoal on downstream macroinvertebrate biodiversity over time

Andrew C. Johnson†*, Monika D. Jürgens†, François K. Edwards†, Peter M. Scarlett†, Helen M. Vincent†, Peter von der Ohe‡

Figure S 1 Dissolved oxygen (DO), ammonium (total ammoniacal nitrogen as N), P and BOD compared to water quality standards (UKTAG 2008) both upstream (us) and downstream (ds) of the Swindon WWTP. *A smaller WWTP, which closed in 1998, was situated between the 6 km and 1 km upstream site.

Figure S 2 Cadmium concentrations and temporal trends for sewage effluent and downstream (ds) of the Swindon WWTP. Where the concentration was below the limit of quantification (LOQ), the relevant LOQ is plotted as a short horizontal line. These non-detects (38-54% of all measurements at the three sites) were ignored for the regression calculation.

Figure S 3 Records of iron found in the Swindon WWTP effluent.

Figure S 4 Records of dissolved iron found downstream of Swindon WWTP effluent in the River Ray. (Note, that the absolute values are not directly comparable to figure S 3, where total iron was measured, but the trend (or lack of trend) is).

Figure S 5 Flow measured at in the River Ray at Water Eaton (1 km ds of the Seven Bridges site)

| Asselidae 1 km upstream  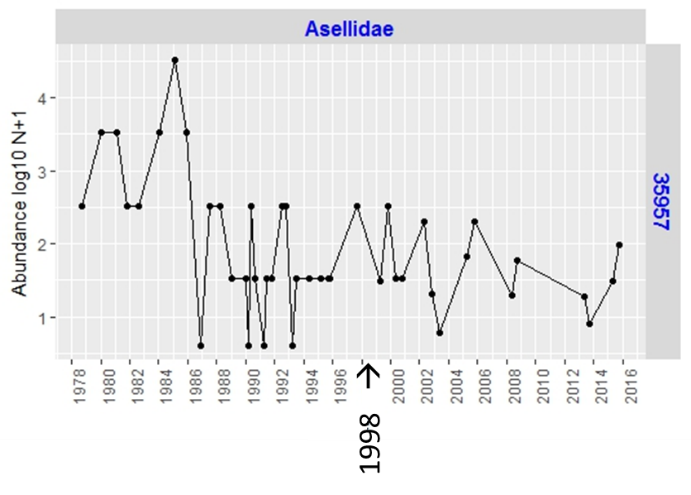 | Asselidae 2 km downstream  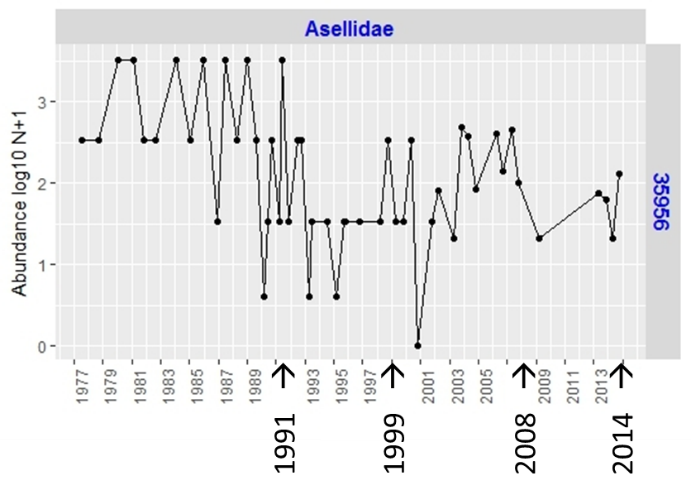 |
| --- | --- |

Figure S 6 The *Assellus* family, surveyed at Morris Street, 1 km upstream, and Moredon Bridge, 2 km downstream of Swindon WWTP. This was the only family for which the highest abundance at the downstream site was associated with the poor pre-1991 water quality period. The arrows indicate the closure of the small Wroughton WWTP upstream of Morris Street in 1998, and major upgrades at the Swindon WWTP in 1991 (change from trickling filter to activated sludge), 1999 (phosphate stripping started), 2008-2014 (beginning and end of the activated charcoal treatment to remove micro-organic contaminants)

| 1 km up-stream | Calopterygidae  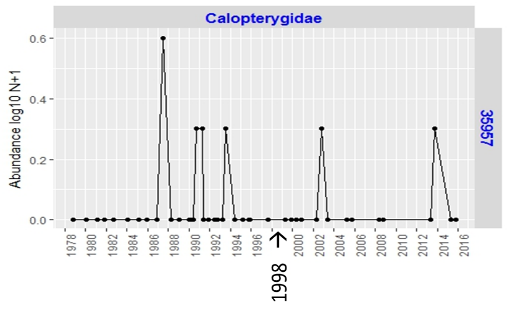 | Hydropsychidae  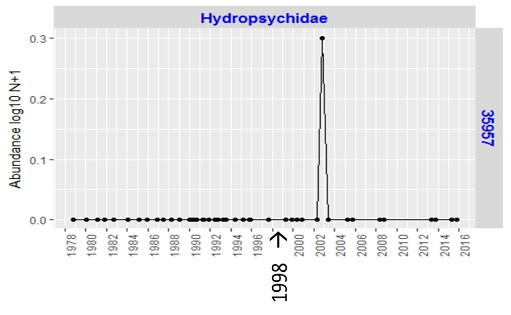 | Limnephilidae  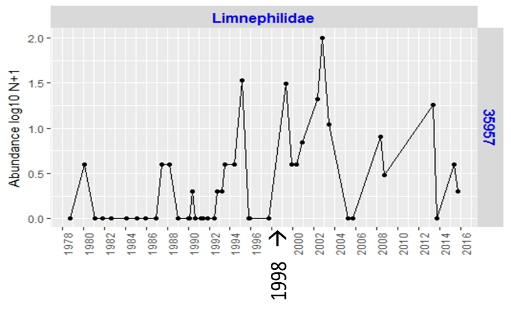 |
| --- | --- | --- | --- |
| 2 km down-stream | 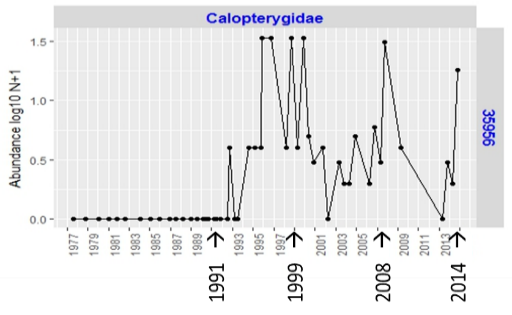 | 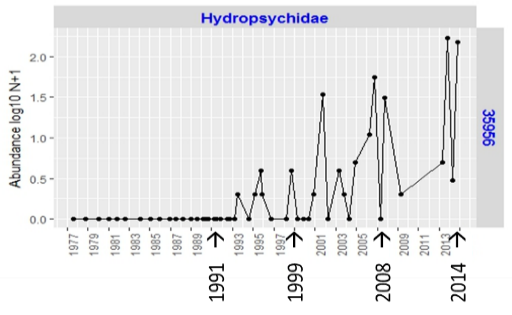 | 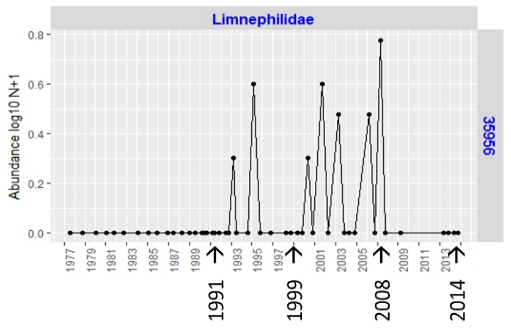 |
| 1 km up-stream | Ceratopogonidae  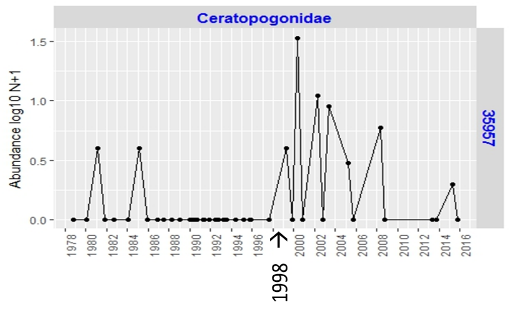 | Dendrocoelidae  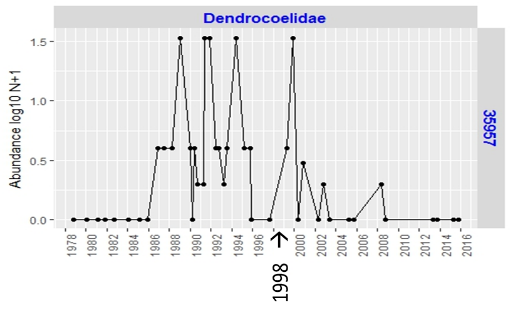 | Planariidae  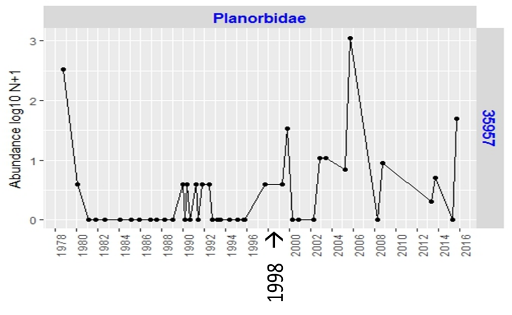 |
| 2 km down-stream | 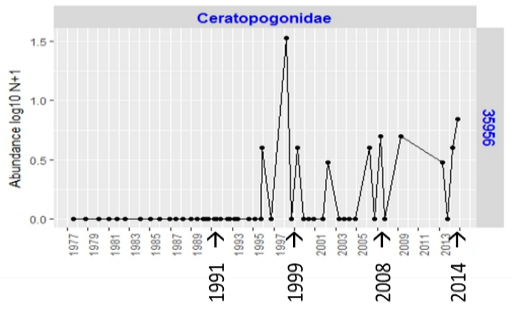 | 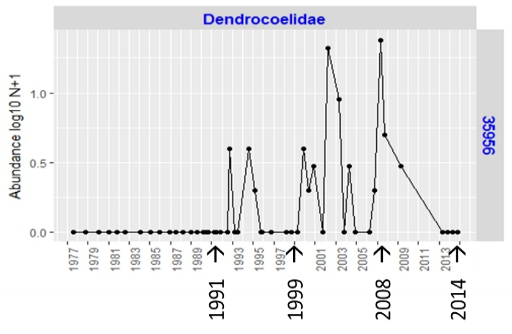 | 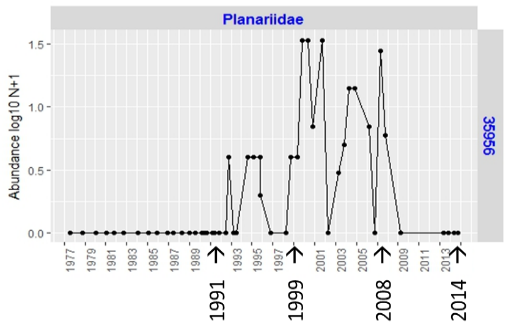 |
| 1 km up-stream | Tipulidae  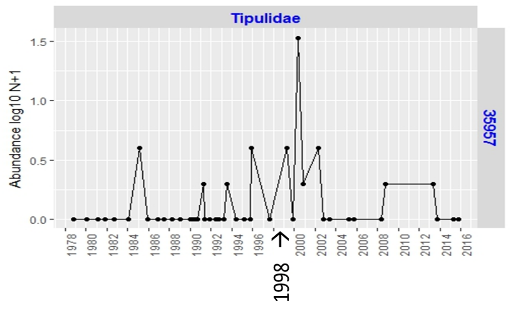 | Ancylidae  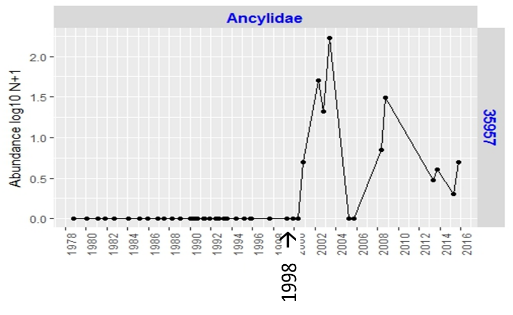 |  |
| 2 km down-stream | 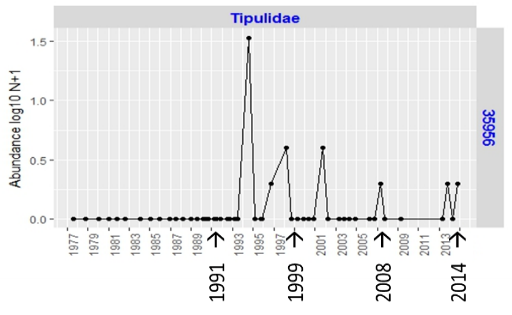 | 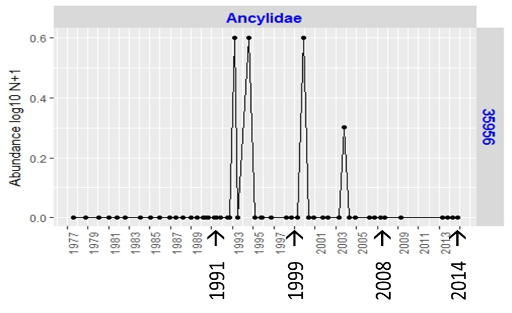 |  |

Figure S 7 Comparing trends in families at Morris St. (upstream Swindon WWTP) and Moredon Br. (downstream Swindon WWTP). These were families which arrived at Moredon Br. **soon after 1991**. The relevance of the highlighted dates is explained in the caption of figure S 6.

| 1 km up-stream | Simuliidae  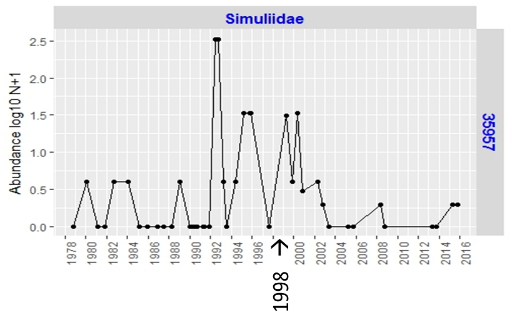 | Hydracarina  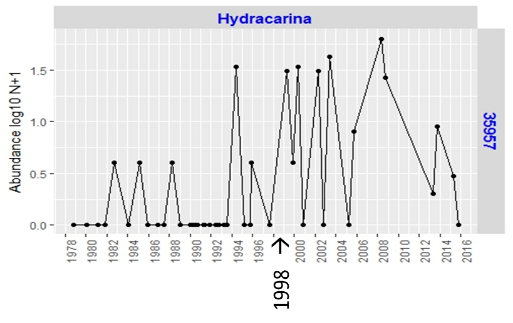 | Hydrobiidae  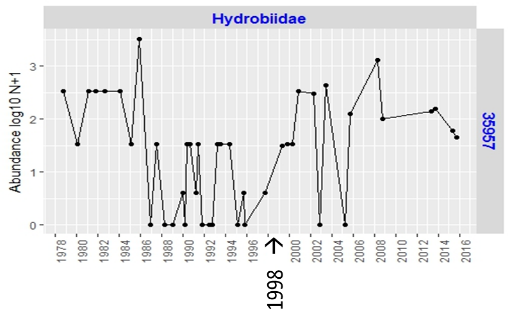 |
| --- | --- | --- | --- |
| 2 km down-stream | 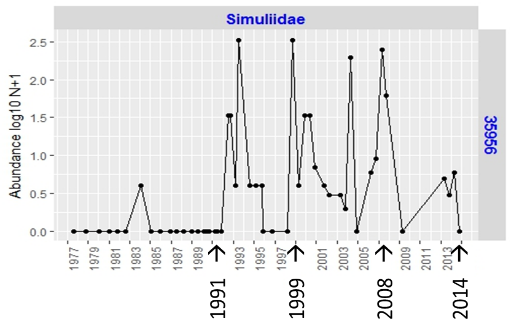 | 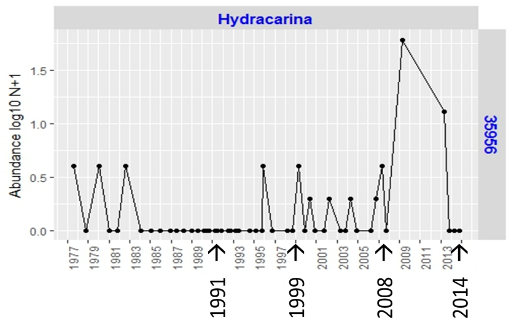 | 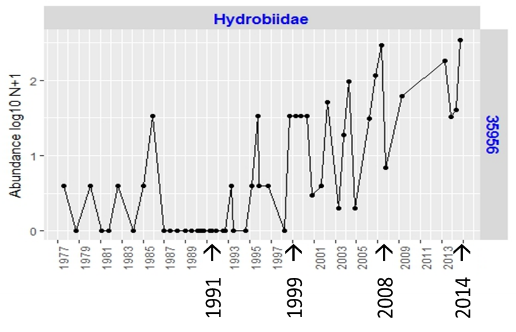 |
| 1 km up-stream | Sphaeriidae  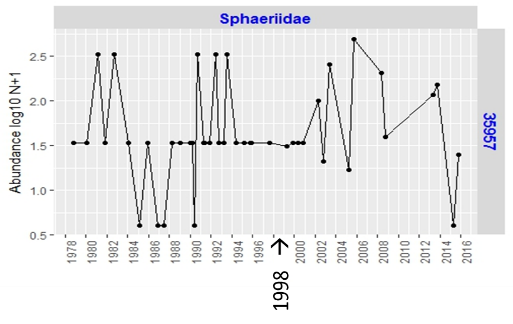 | Elmidae  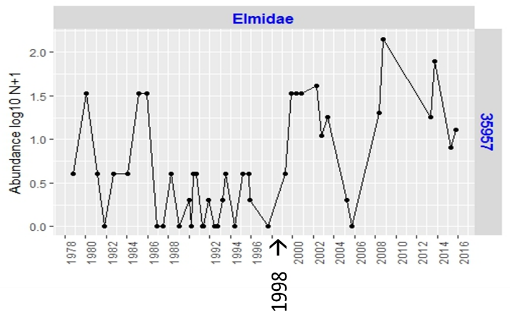 |  |
| 2 km down-stream | 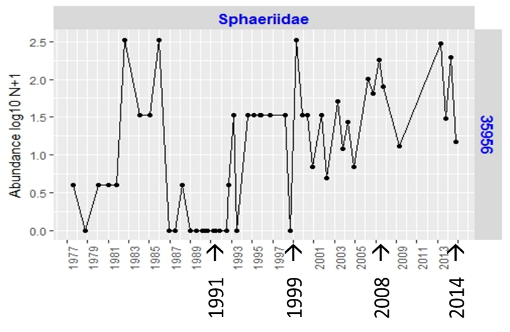 | 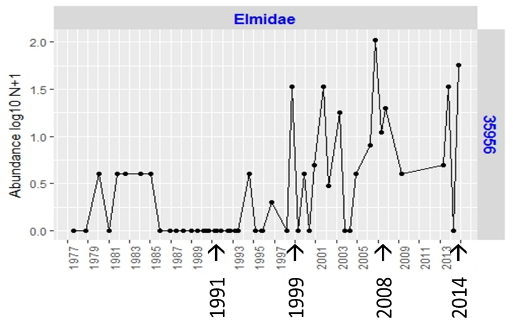 |  |

Figure S 8 Comparing trends in families at Morris St. (1 km upstream Swindon WWTP) and Moredon Br. (2 km downstream Swindon WWTP). These were families which were **absent for a period** at Moredon Bridge before 1991. The relevance of the highlighted dates is explained in the caption of figure S 6.

| 1 km up-stream | Acroloxidae  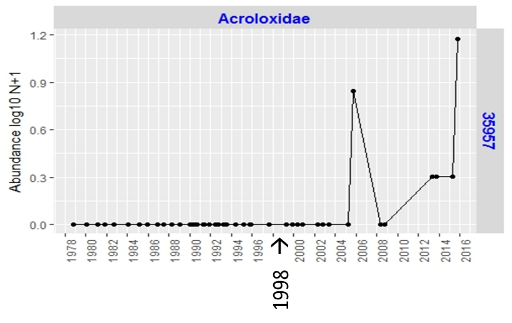 | Crangonyctidae  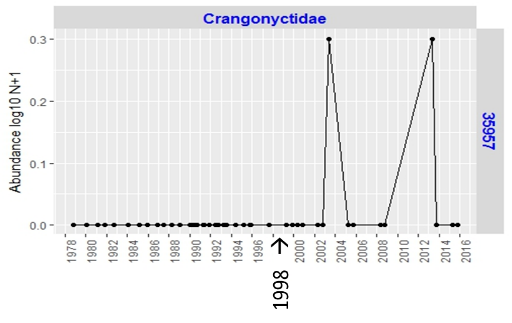 | Dugesiidae  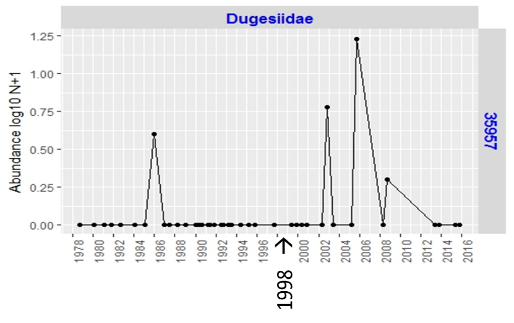 |
| --- | --- | --- | --- |
| 2 km down-stream | 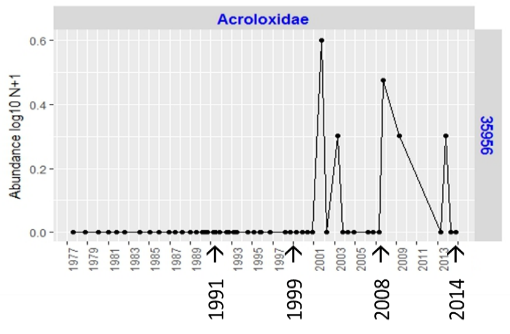 | 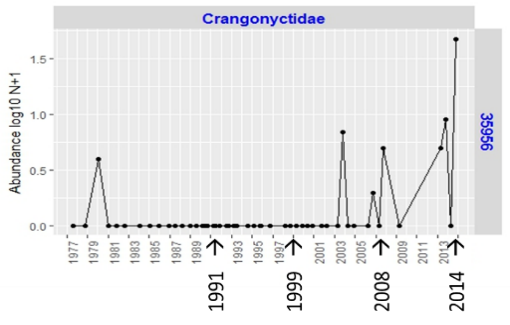 | 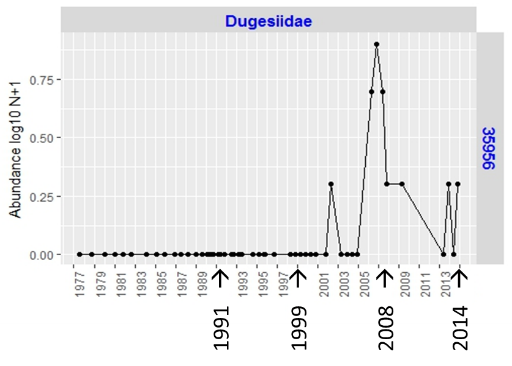 |
| 1 km up-stream | Hydroptilidae  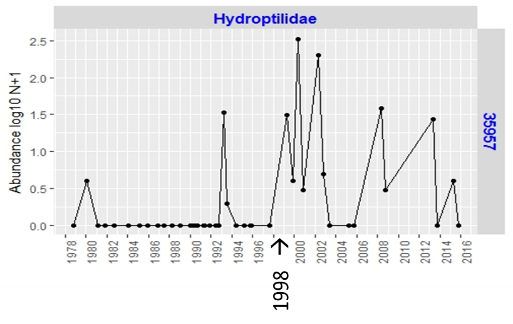 | Leptoceridae  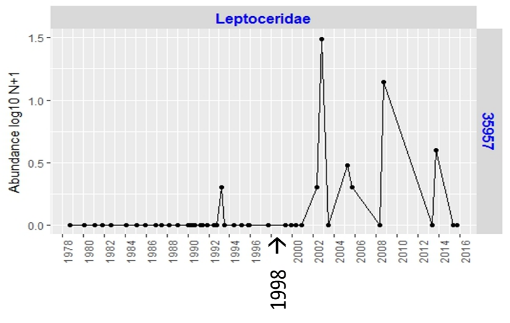 | Empididae  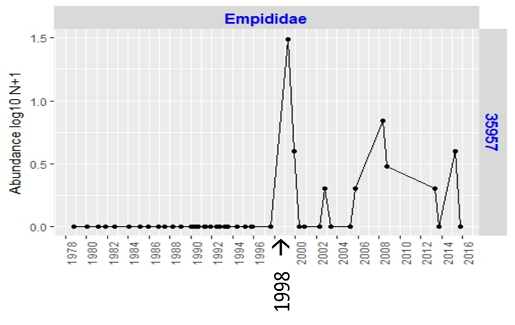 |
| 2 km down-stream | 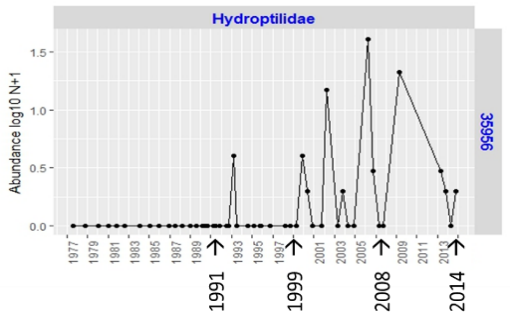 | 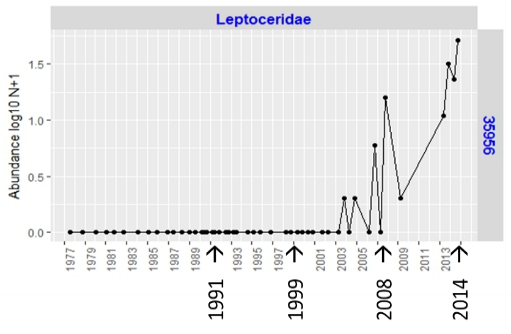 | 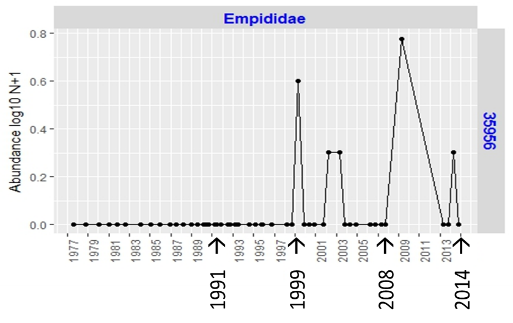 |

Figure S 9 Comparing trends in families at Morris St (upstream Swindon WWTP) and Moredon Br (downstream Swindon WWTP). These were families which mostly arrived at Moredon Bridge in **the late 1990s-2000 period**. The relevance of the highlighted dates is explained in the caption of Figure S 6.

| 1 kpm up-stream | Baetidae  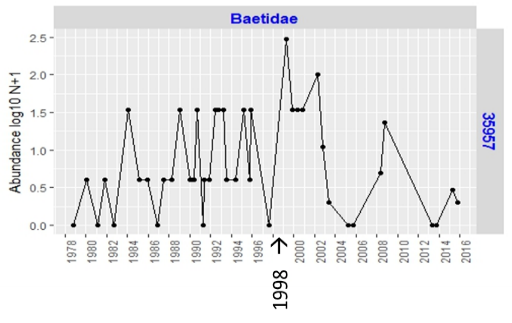 | Leptophlediidae  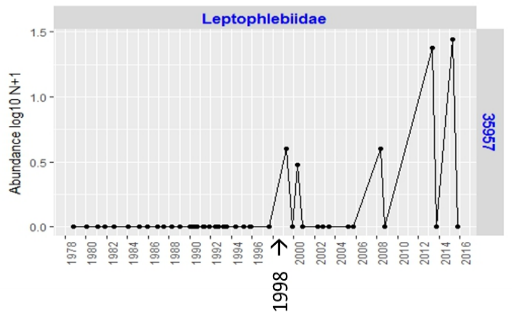 | Chironomidae  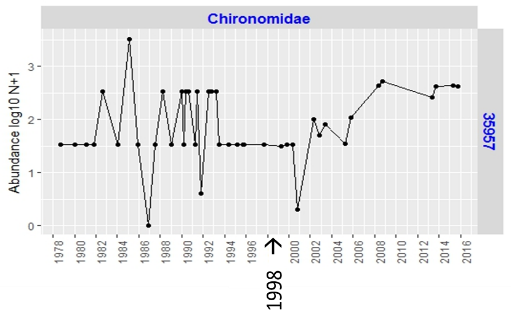 |
| --- | --- | --- | --- |
| 2 km down-stream | 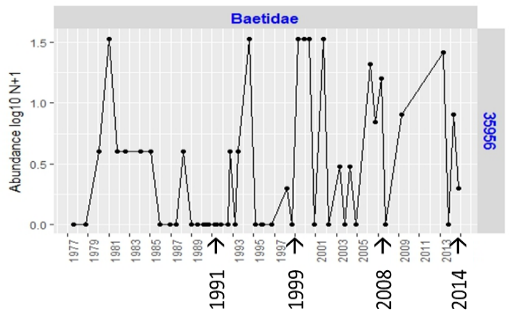 | 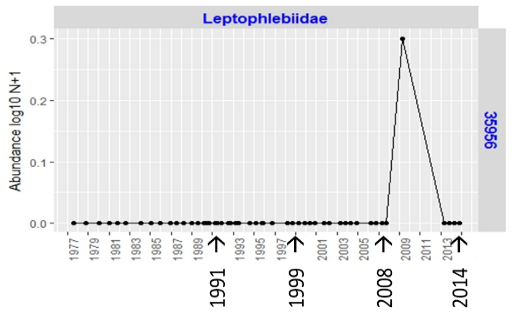 | 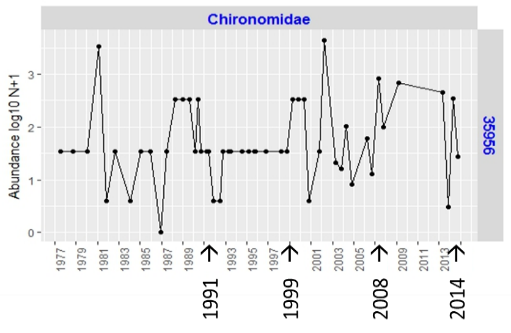 |
| 1 kpm up-stream | Psychodidae  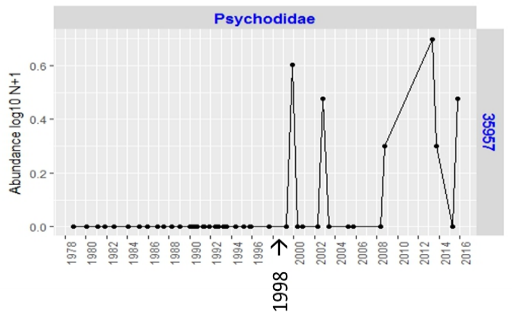 | Dytiscidae  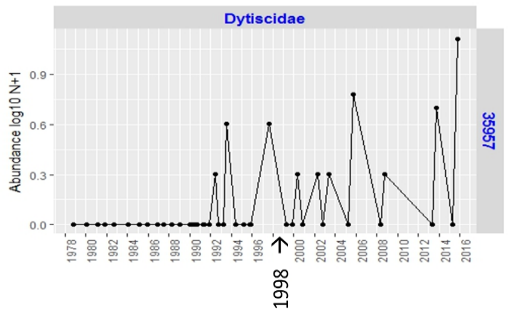 | Haliplidae  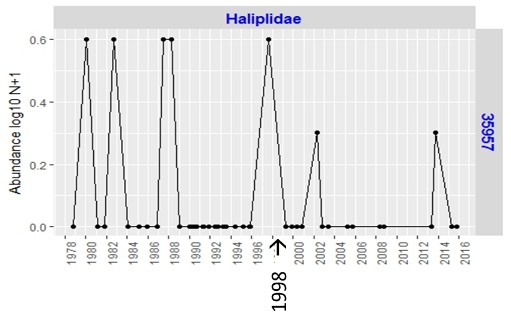 |
| 2 km down-stream | 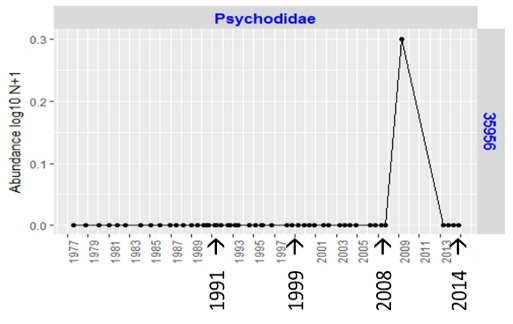 | 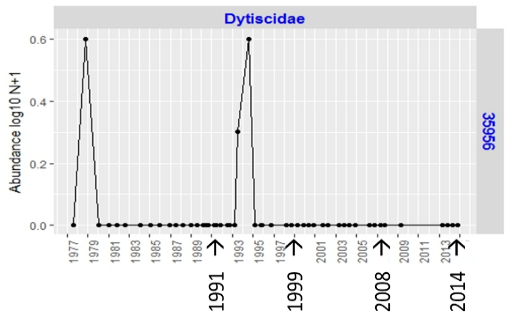 | 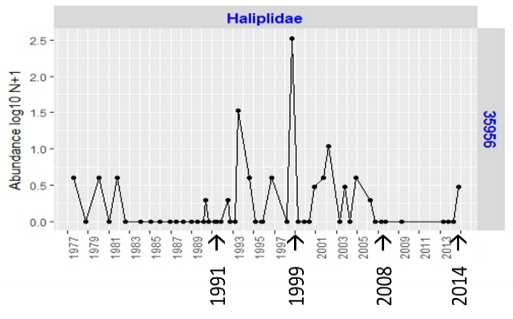 |
| 1 kpm up-stream | Hydrophilidae  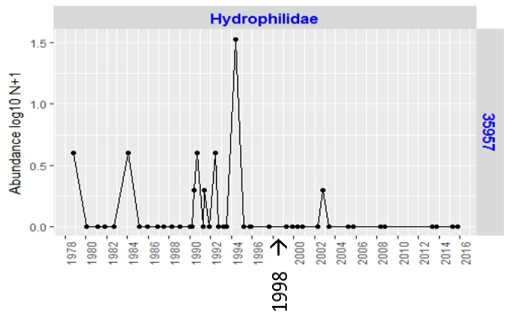 | Lymnaeidae  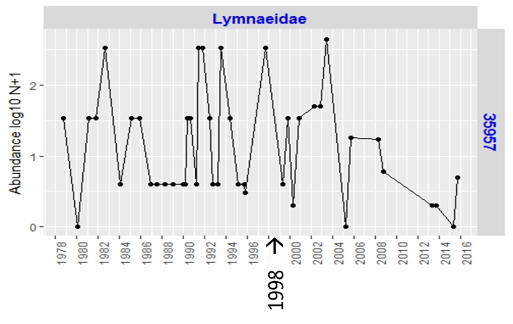 | Physidae  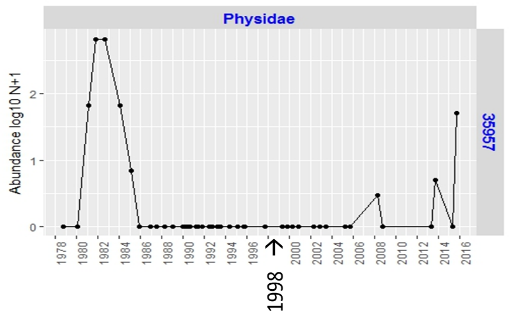 |
| 2 km down-stream | 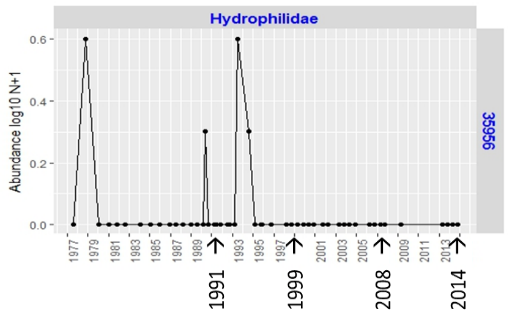 | 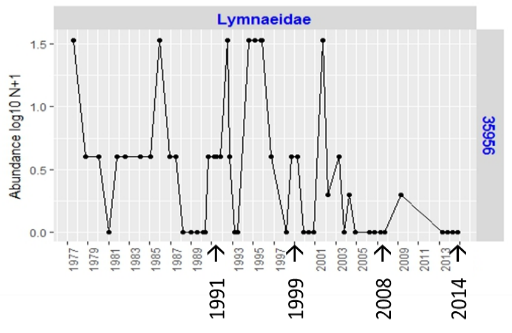 | 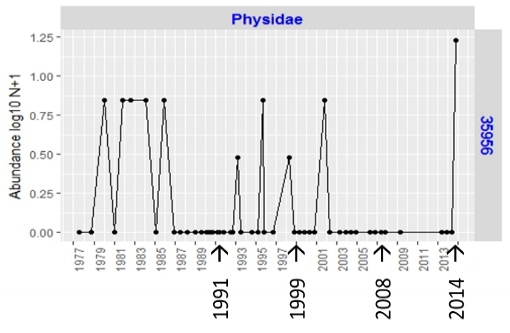 |

**Figure S10 continued on next page**

**Figure S10 continued from previous page**

| 1 kpm up-stream | Planorbidae  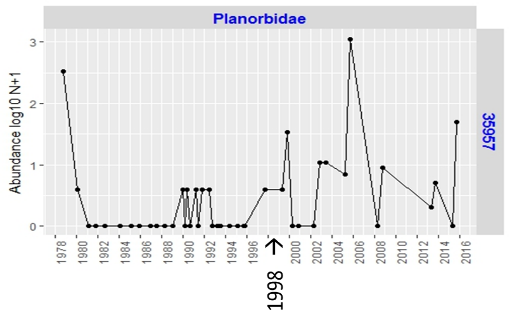 | Gammaridae  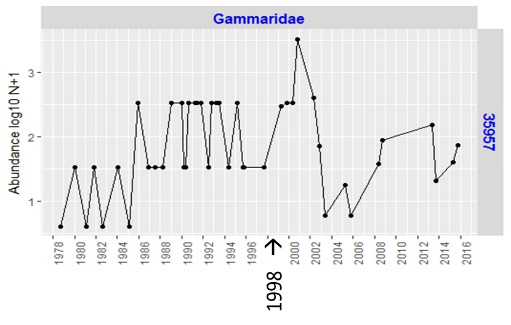 | Glossiphoniidae  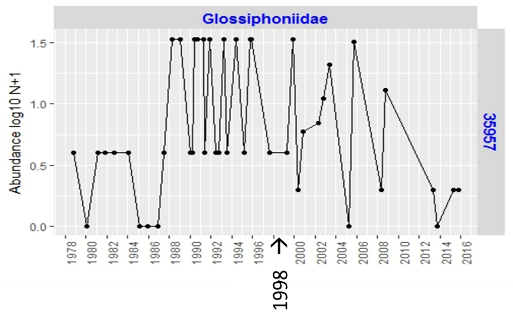 |
| --- | --- | --- | --- |
| 2 km down-stream | 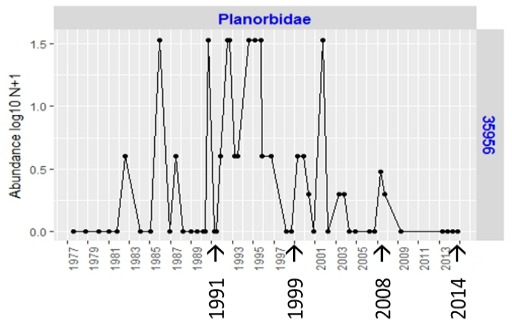 | 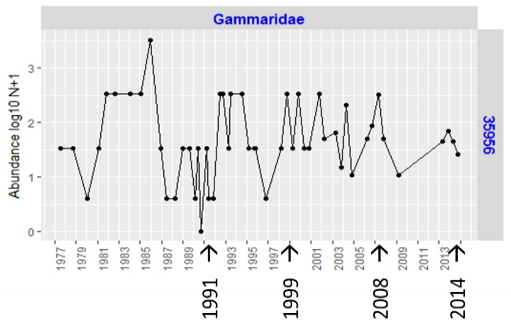 | 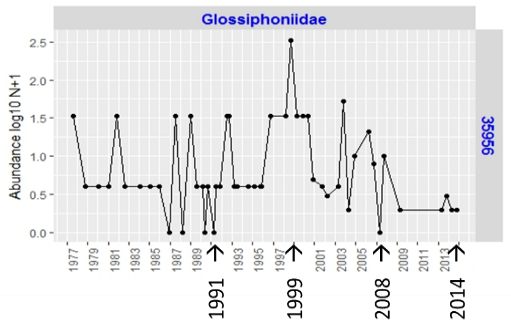 |
| 1 km up-stream | Psychomiidae  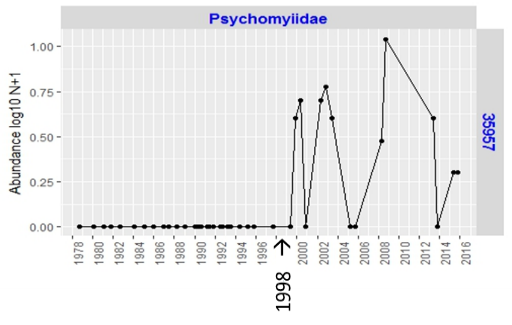 | Erpobdellidae  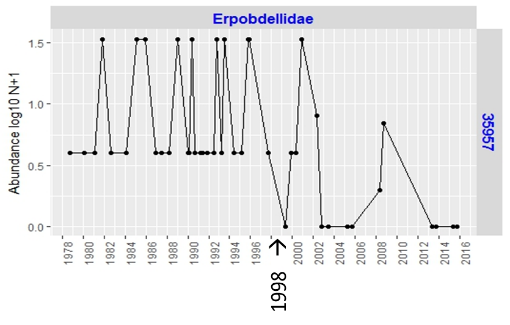 |  |
| 2 km down-stream | 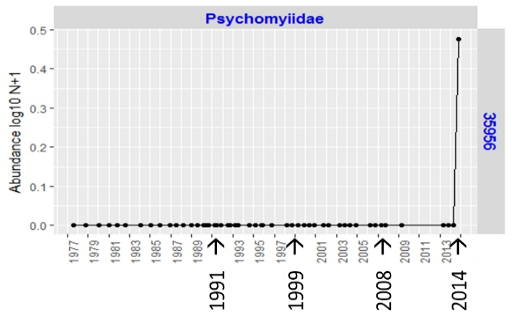 | 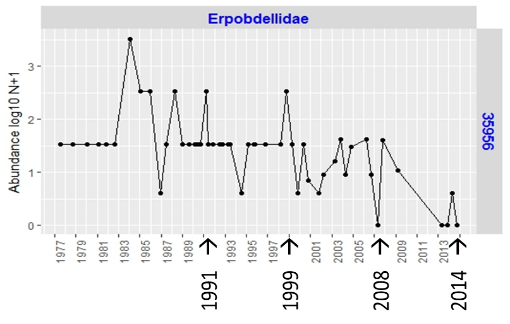 |  |

Figure S 10 Comparing trends in families at Morris St (upstream Swindon WWTP) and Moredon Br (downstream Swindon WWTP). These were families whose presence or absence at Moredon Bridge seemed **unconnected** to the changes at Swindon WWTP. The relevance of the highlighted dates is explained in the caption of Figure S 6.
